# Exploring the frame effect

**DOI:** 10.1167/jov.22.12.5

**Published:** 2022-11-02

**Authors:** Patrick Cavanagh, Stuart Anstis, Matteo Lisi, Mark Wexler, Marvin R. Maechler, Bernard Marius ’t Hart, Mohammad Shams-Ahmar, Sharif Saleki

**Affiliations:** 1 Department of Psychology, Glendon College, Toronto, Ontario, Canada; 2 Centre for Vision Research, York University, Toronto, Ontario, Canada; 3 Department of Psychology, University of California at San Diego, La Jolla, CA, USA; 4 Department of Psychology, Royal Holloway, University of London, London, UK; 5 INCC and CNRS, Université de Paris, Paris, France; 6 Department of Psychological and Brain Sciences, Dartmouth College, Hanover, NH, USA

**Keywords:** motion-induced position shift, frame effect, position

## Abstract

Probes flashed within a moving frame are dramatically displaced (Özkan, Anstis, ‘t Hart, Wexler, & Cavanagh, 2021; Wong & Mack, 1981). The effect is much larger than that seen on static or moving probes (induced motion, Duncker, 1929; Wallach, Bacon, & Schulman, 1978). These flashed probes are often perceived with the separation they have in frame coordinates—a 100% effect (Özkan et al., 2021). Here, we explore this frame effect on flashed tests with several versions of the standard stimulus. We find that the frame effect holds for smoothly or abruptly displacing frames, even when the frame changed shape or orientation between the end points of its travel. The path could be nonlinear, even circular. The effect was driven by perceived not physical motion. When there were competing overlapping frames, the effect was determined by which frame was attended. There were a number of constraints that limited the effect. A static anchor near the flashes suppressed the effect but an extended static texture did not. If the probes were continuous rather than flashed, the effect was abolished. The observational reports of 30 online participants suggest that the frame effect is robust to many variations in its shape and path and leads to a perception of flashed tests in their locations relative to the frame as if the frame were stationary. Our results highlight the role of frame continuity and of the grouping of the flashes with the frame in generating the frame effect.

## Introduction

Vision has been shown to encode the motions and positions of objects relative to the frames that surround them (Duncker, 1929; Johansson, 1950). Frames can change what we judge to be “up” (Asch & Witkin, 1948; Morgan, Grant, Melmoth, & Solomon, 2015) and what direction we think is straight ahead (Matin & Fox, 1989; Roelofs, 1935). A moving frame can alter the sense of our own motion (Warren, 1895) or that of an object within the frame (Duncker, 1929; Johansson, 1950; Wallach 1959). However, this earlier literature on frame effects typically examined static (Duncker, 1929) or continuously moving probes (Wallach et al., 1978). In contrast, moving frames give far larger effects for flashed rather than continuous probes (Özkan et al., 2021; Wong & Mack, 1981). The illusory offsets can be as large as the frame's displacement, as if the flashes were seen in the frame's coordinates and the frame were not moving. Moreover, unlike continuous probes, flashed probes do not appear to move. Johansson (1950) had proposed a process of motion decomposition for groups of elements in motion. This decomposition would extract a common motion vector shared by all the elements and leave the differences from the group vector as relative motion. But flashed tests do not appear to have either the common or relative motion. Instead of a motion decomposition, we get a position decomposition and the effects are dramatic. This perception of the flash locations relative to the stabilized frame, rather than to their actual screen coordinates, may be linked to visual stability where the displacement of the entire visual scene acts as a moving frame that stabilizes position as the eyes move.

In this paper, we explore several variations of the effect of moving frames on flashed probes. Observational reports were collected from 30 online participants for 20 versions of the stimulus that varied the nature of the frame, the properties of its motion, and the grouping of the flashes with the frame. In most cases, the results are readily visible in the 20 movies. The observations we collected were simple subjective reports (e.g. yes, I see it) rather than controlled parametric tests, but they help identify which stimuli give strong effects and which give weak, ambiguous, or no effects. These results will then guide the selection of more controlled, future testing.

## Methods

### Participants

The experiment was conducted online. Thirty participants were recruited by email sent to vision laboratories across the world. Six of the eight co-authors also participated. The protocols for the study were approved by the York University Review Board in accordance with the principles of the Declaration of Helsinki (WMA, 2013). The consent form was part of the recruitment email and, if the participant consented, they then clicked on the link leading to the test videos.

### Stimuli and procedure

The experiment consisted of a set of 21 movies presented to the participants in their web browser, accessed here at https://cavlab.net/Demos/FrameExperiment. Following the comments of several participants and a reviewer that the instructions and stimulus were hard to interpret for one movie (#13 in the experimental series), this movie was excluded from further analysis. The following results sections cover the remaining 20 tests and present the same movies that participants saw, although the order is different in a few cases. Each movie had a frame or background that moved back and forth at 0.9 Hz and two flashes, one red and one blue that appeared at the moment the motion paused and reversed direction. There were four exceptions: in the section “Is the frame effect due to displacement or motion?” the movie had two red flashes; in Movie 11, the frame's motion had no reversal; also in Movie 11, the motion did not pause while the flashes were present; and, finally, in Movie 20, the probes are present continuously in the first half of the movie. In 19 of the movies, the frame motion was horizontal and the flashes were vertically aligned. For these movies, the flashes were also vertically separated center-to-center by approximately twice the diameter of the flashed discs. This vertical offset could then combine with any illusory horizontal offset to create a noticeable angle between the upper and lower flashes that was more easily detected than the pure horizontal offset that would be generated for superimposed flashes. In one of these 19 movies (Movie 13), the horizontally moving frame was skewed and the offset could be horizontal or vertical. Finally, Movie 4 had vertical frame motion and an illusory offset, if there were any, would be vertical. On the test pages, there was a brief preamble above the movie for the participants to read before viewing the movie and the details of these introductory comments will be summarized in each result section when they are informative. The question for each test was presented below the movie and the movie looped continuously until the observer had chosen a response and clicked to move to the next test. For most of the movies, the participants were asked whether the red flashed disc was seen to the right of the blue and they responded with a 4-point scale (1. Yes; 2. Yes, after a while; 3. Not much; and 4. No). Other responses were specific to particular movies and these will be described in each section where appropriate. Observers were also asked to attach any comments they had on anything they noticed, although very few did so. All of these details can be seen by accessing the experiment itself at https://cavlab.net/Demos/FrameExperiment. The observers returned their responses by email. The experiment took 10 to 15 minutes. The experiment, data, consent forms and analyses are available at https://osf.io/b2vu8/.

### Analysis

The responses of the participants are combined and reported with the question and response choices for each of the 20 movies.

## Results

### Where frames work

#### The basic frame effect

Here is the basic frame effect with an outline square moving left and right and a probe flashing at each reversal (Movie 1). The red and blue discs are *always* vertically aligned—notice the green line—but when the line disappears, a large offset may appear with red to the right of the blue. All observers reported this offset (Table 1). The green line in these movies was not present in the experimental movies. Observers were instructed not to stare at or fixate on the flashes. The same holds here for these demonstrations. Please avoid fixating on the flashes because, for some, this may reduce or eliminate the effects.

**Movie 1.** Frame Effect.

**Table 1.** Responses (out of 30).

		
Does the red flash appear to the right of the blue flash?	30	Yes.
	0	Yes, but only after a while.
	0	Not so much.
	0	No.

#### Apparent motion

Here is the same basic frame effect but now with a single step in the displacement—apparent motion (Movie 2). The red and blue discs here are again *always* vertically aligned—notice the green line—but when the line disappears, the offset may appear with red to the right of the blue. Seventy-six percent of the observers reported seeing this offset immediately or after some delay (Table 2). Seventeen percent saw no offset.

**Movie 2.** Apparent motion Frame effect.

**Table 2.** Responses (out of 30).

		
Does the red flash appear to the right of the blue flash?	19	Yes.
	4	Yes, but only after a while
	2	Not so much.
	5	No.

#### Second-order motion

**Figure 1.**
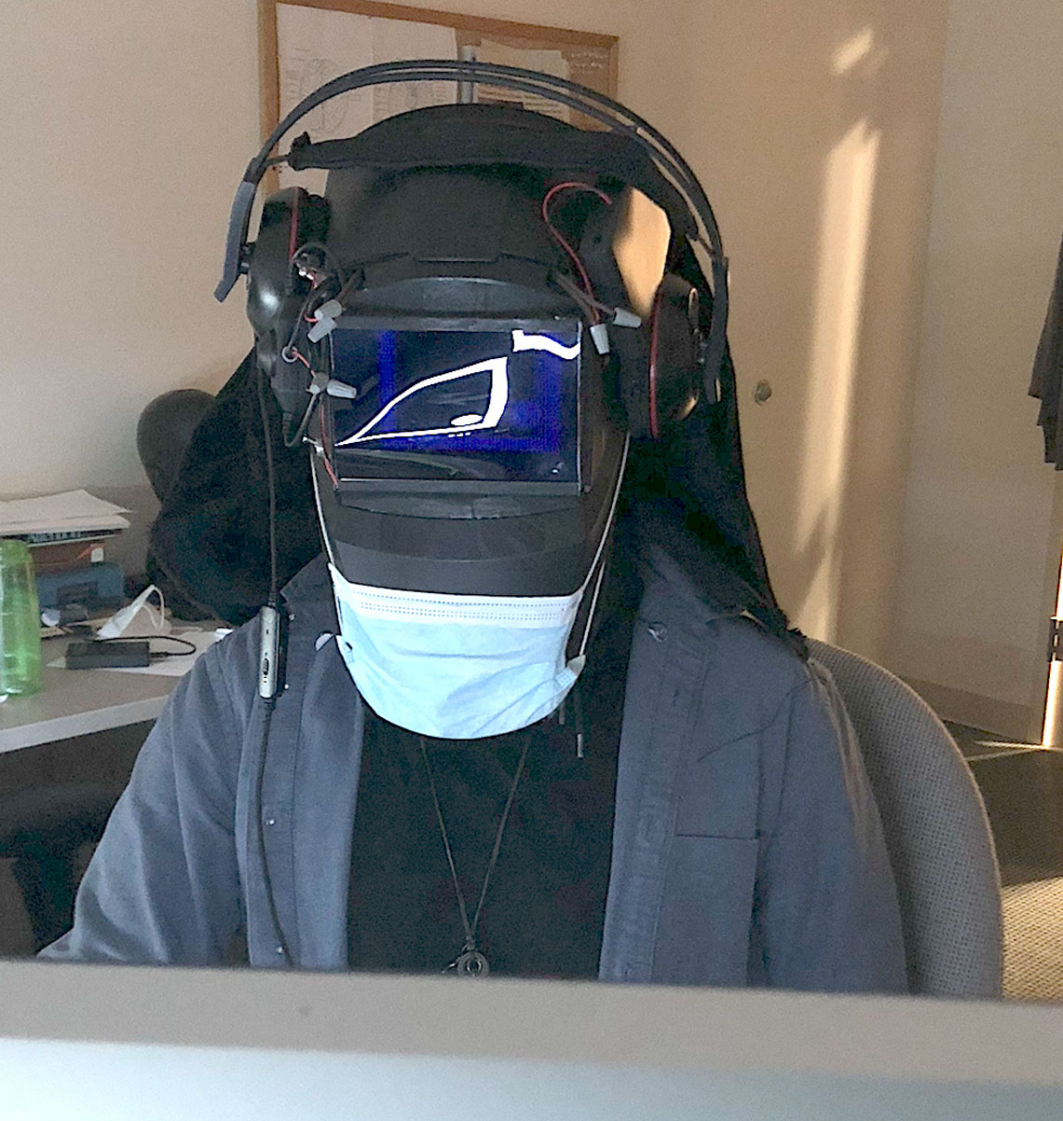
Viewing stimuli with the equiluminizing helmet. The screen display is reflected off the front blue filter. See Connolly, Connolly, Cleary, Herman, and Cavanagh (2017).

Here, the frame is a second-order shape defined by flickering dots against the surrounding steady random dots (Cavanagh & Mather, 1989), there is no difference in mean luminance between the frame and the background (Movie 3). A pilot test was also run with one observer where the frame was defined by equiluminous color using a specialized helmet (Figure 1). The observer reported a strong frame effect. In the second-order motion demonstration below, the red and blue discs are again *always* vertically aligned—notice the green line—but when the line disappears, a large offset may appear with red to the right of the blue. Ninety-seven percent of the observers saw this offset immediately or with some delay (Table 3). Three percent did not.

**Movie 3.** Second-order motion frame effect.

**Table 3.** Responses (out of 30).

		
Does the red flash appear to the right of the blue flash?	27	Yes.
	2	Yes, but only after a while
	0	Not so much.
	1	No.

#### Two frames at once

Here, two frames move in opposite directions with all four flashes aligned horizontally (Movie 4). Ninety-seven percent of the observers reported the vertical offsets in both frames immediately or with some delay (Table 4). The preamble for this test showed how the offset, if any, should be judged as the vertical separation seen between the flashes within each frame and not between the frames. The result here shows that the frame effect is not due to eye movement artifacts, as any pursuit would affect the separations of the perceived flash locations in opposite directions in the two frames.

**Movie 4.** Double frame effect.

**Table 4.** Responses (out of 30).

		
Are red and blue separated vertically in each frame?	28	Yes.
	1	Yes, but only after a while
	1	Not so much.
	0	No.

#### Is the frame effect due to displacement or motion?

We find that both the displacement and the motion generate position offsets, although the effect of pure motion is much smaller than that of the standard frame effect (Cavanagh, MacLeod, & Anstis, 2021). Pure motion is tested here using reverse apparent motion (Anstis, 1970) and the stimulus offers no discernible landmarks to carry displacement information. The random dot field moves rigidly one way, reversing contrast on each step, but the perceived motion goes in the opposite direction (Movie 5). Ninety-three percent of the observers reported that the flashes, which are always vertically aligned, appear displaced in agreement with the perceived direction and not the physical displacement (Table 5). Three percent reported not seeing any shift. To demonstrate the step and contrast reversal, a second movie (Movie 5a) shows a small part of the test movie slowed down 30 times and magnified in size.

**Movie 5.** Reverse apparent motion.

**Table 5.** Responses (out of 30).

		
Is the top red flash to the right of the bottom one?	19	Yes.
	9	Yes, but only after a while
	1	Not so much.
	1	No.

**Movie 5a.** A close up of the reverse apparent motion Movie 5 above at 1 frame per second to show how the pattern shifts left and reverses contrast on each step. The green star moves along with the pattern to help track the pattern's displacement. At full speed, this produces an impression of rightward motion, opposite to the direction of the physical motion.

#### What moving backgrounds can produce the frame effect?

Any moving background appears to be sufficient, from natural scenes to outline squares and random dots. Even two discs moving in tandem can be effective (Movie 6). The red and blue discs here are always vertically aligned, as shown at the beginning by the green line, but red may appear shifted to the right of the blue once the line disappears. Ninety percent reported the offset immediately or with some delay, 7% did not see it (Table 6).

**Movie 6.** Two discs as frame.

**Table 6.** Responses (out of 30).

		
Does the red flash appear to the right of the blue flash?	25	Yes.
	2	Yes, but only after a while
	1	Not so much.
	2	No.

#### Can the background change while moving?

The background can distort and rotate while still preserving the frame effect. It appears sufficient that some “thing” has displaced. It may change as it displaces. This may be related to the tolerance of apparent motion to shape and feature changes between the first and second position. Here, the shape is changing during the motion but once the green line fades, the red disc appears to the right of the blue, even though they are vertically aligned (Movie 7). Ninety-three percent reported seeing the offset immediately or with some delay, 3% did not see it (Table 7).

**Movie 7.** Non-rigid frame.

**Table 7.** Responses (out of 30).

		
Does the red flash appear to the right of the blue flash?	26	Yes.
	2	Yes, but only after a while
	1	Not so much.
	1	No.

#### Can the background rotate while moving?

Here, the shape rotates while displacing (Movie 8). The effect (red to the right of the blue) may be reduced for some observers. Seventy percent reported seeing the offset immediately or with some delay, 30% saw little or no effect (Table 8).

**Movie 8.** Rotating frame.

**Table 8.** Responses (out of 30).

		
Does the red flash appear to the right of the blue flash?	15	Yes.
	6	Yes, but only after a while
	4	Not so much.
	5	No.

#### Does the displacement have to be linear?

The backgrounds can follow nonlinear paths while still preserving the frame effect, suggesting that it is driven solely by the displacement between initial and final locations when the two flashes appear. Here is a path that follows a semicircular arc (Movie 9). Eighty-seven percent reported seeing the effect immediately or with some delay, 7% did not see it (Table 9).

**Movie 9.** Semicircular path.

**Table 9.** Responses (out of 30).

		
Does the red flash appear to the right of the blue flash?	24	Yes.
	2	Yes, but only after a while
	2	Not so much.
	2	No.

#### Does the displacement have to be linear?

**Figure 2.**
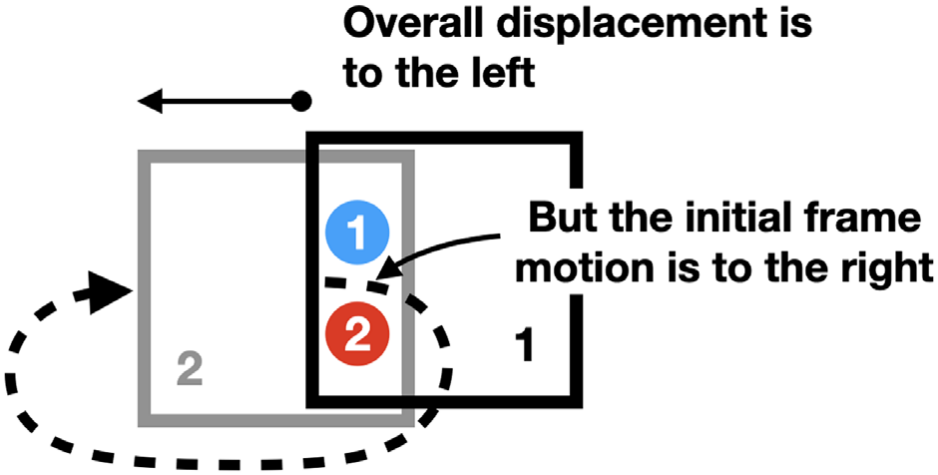
Direction reversing path.

Does the flashed probe shift because it is pulled by the initial motion of the frame or is its shift a result of the frame's overall path? We can test this with a nonlinear path that starts out in the direction opposite to the final displacement (Figure 2) like the one below (Movie 10). If the shift is caused by the initial motion, the red will appear to the left of the blue. If it is caused by the overall displacement, the red will appear to the right of the blue. The outcome was equivocal though, 43% reported seeing red to the right, but 47% reported no offset (Table 10). This movie might have been placed in the second group (2. Where frames do not work) but it also fits here as an extension of the preceding case of less extreme nonlinear displacement.

**Movie 10.** Extreme nonlinear path.

**Table 10.** Responses (out of 30).

		
Does the red flash appear to the right of the blue flash?	8	Yes.
	5	Yes, but only after a while
	3	Not so much.
	14	No.

#### Does the displacement have to have transient reversals?

The backgrounds can follow a continuous circular path with no reversal transient while still preserving the frame effect (Movie 11). Unlike the other movies, the frame here continues to move while the flashes are present. Eighty-three percent saw the offset immediately or after some delay in this movie, 17% saw little or no effect (Table 11).

**Movie 11.** Continuous path.

**Table 11.** Responses (out of 30).

		
Does the red flash appear to the right of the blue flash?	18	Yes.
	7	Yes, but only after a while
	3	Not so much.
	2	No.

#### Attention: What happens when there are two frames?

When two frames move in opposite directions, the flashed discs may initially appear vertically aligned, as they are (Movie 12). But **attention** to the light or dark frame can make the red dot appear to the left of the blue (attend to light frame) or to the right (attend to the dark frame). The preamble for this movie suggested that observers could attend to one frame or the other (without saying how) and indicated that this might affect the relative positions of the red and blue flashes. Sixty-three percent saw the two flash offsets reverse when they switched attention, 23% saw no offset, and 13% saw an offset but could not reverse it (Table 12).

**Movie 12.** Overlapping frames, attend to one or the other.

**Table 12.** Responses (out of 30).

		
Is the red flash left of blue at times but right of it at other times?	19	Yes.
	4	I see a separation, but it never reverses.
	0	Not so much.
	7	No.

#### Is the frame effect driven by global or local motion?

Here is the first example to demonstrate that the frame effect is determined by the global, perceived motion not by the local, physical motion (Movie 13). Here, in an angled parallelogram, the perceived motion is up and down even though the local motion of the nearest contours is left and right. When viewing the whole shape, the two flashes shift apart vertically, consistent with the perceived, global motion. However, when viewing is restricted to a horizontal aperture, the left-right motion dominates and the red and blue may shift apart horizontally. Indeed, all observers saw the offset as vertical before the occluders were present, then horizontal after they appeared (Table 13).

**Movie 13.** Aperture frame.

**Table 13.** Responses (out of 30).

		
Is the blue flash first above red, but in the restricted view more left of red?	28	Yes.
	2	Yes, but only after a while
	0	Not so much.
	0	No.

#### Is the frame effect driven by global or local motion?

In this second example, an occluded diamond shifts left and right (Lorenceau & Shiffrar, 1992). With gaze near the flashing discs, the motion of the white contours appears to be up and down and there may be no shift of the two flashes (Movie 14). However, with gaze directed away to the left or right (observers were instructed to try fixating outside the page), a left-right motion may be seen and the two flashes should then shift apart horizontally. Sixty-seven percent of the observers did report the offset when looking peripherally, and 33% saw little or no offset no matter where they looked (Table 14).

**Movie 14.** Occluded diamond frame.

**Table 14.** Responses (out of 30).

		
Does red appear to the right of blue when looking outside the movie frame?	12	Yes.
	8	Yes, but only after a while
	7	Not so much.
	3	No.

### Where frames do not work

#### Frames do not work when there is a nearby spatial anchor

Here, there is initially one vertical line (Movie 15). Its presence anchors the locations of the two flashes and 70% of the observers report little or no shift indicating that the nearby static anchor has suppressed the effect of the frame, perhaps because it provides a strong position reference. However, if more lines are added, the frame effect returned for 43% of observers, suggesting that the original line lost its position signal as it became an indistinguishable part of a texture (Table 15). Interestingly, 30% of observers already saw an offset between the two flashes when only one line was present, and they saw the same offset when all the lines were present. Seventeen percent saw no offset in either configuration.

**Movie 15.** Superimposed static line, then grid.

**Table 15.** Responses (out of 30).

		
Is red to the right of blue *only* after the extra lines appear?	13	Yes, only after.
	9	Red right of blue at all times.
	3	Not so much before or after.
	5	Nothing.

#### But static textures do not act as spatial anchors

The previous example showed that not all nearby static features will suppress the frame effect, at least if they form a regular texture. Here is a second counterexample where the arrangement of static features is random (Movie 16). The frame effect still works for 90% of the observers despite the nearby stationary spots that the texture provides (Table 16). This shows that the texture does not have to be uniform to lose its position input to the flash judgments. Only 3% of observers reported no offset here.

**Movie 16.** Static random texture.

**Table 16.** Responses (out of 30).

		
Does the red flash appear to the right of the blue flash?	27	Yes.
	0	Yes, but only after a while
	2	Not so much.
	1	No.

#### When the frame rotates around the flashes

If the frame rotates around the tests, here by 180 degrees, the frame effect appears to be suppressed for many observers (43%)—red is not seen to the right of the blue (Movie 17, Table 17). However, some observers (27%) did see an offset here. Notice that the start and end positions of the frame are the same as in the original effect. However, the path between the two end points is quite different, with a rotation around the flash locations rather than a translation across them. Possibly, the continuous presence of one edge near the flashes acts as an anchor, at least for some observers.

**Movie 17.** One hundred eighty degrees frame rotation.

**Table 17.** Responses (out of 30).

		
Does the red flash appear to the right of the blue flash?	5	Yes.
	3	Yes, but only after a while
	9	Not so much.
	13	No.

#### When the frame rotates around the flashes

Here is a similar case but with 270 degrees of rotation (Movie 18). Now, 70% see no offset and the rest, not much (Table 18). If anything, red may be seen to the left of the blue as two observers noted in their comments. The start and end position of the frame are again the same as in the original effect, showing that it is not only the end positions that count.

**Movie 18.** Two hundred seventy degrees frame rotation.

**Table 18.** Responses (out of 30).

		
Does the red flash appear to the right of the blue flash?	0	Yes.
	0	Yes, but only after a while
	9	Not so much.
	21	No.

#### When the frame flips in 3D

Instead of rotation in the plane, the frame now flips out of the plane (Movie 19). Here, 93% of observers report little or no effect, and only 7% see an effect (Table 19). In fact, you might see red to the left of the blue, as one observer noted in their comments, suggesting that the edge that stays near the flashes serves as a frame or anchor and positions are seen relative to it rather than relative to the flipping frame: red is to the left of that edge when it flashes, and blue to its right when it flashes. The start and end positions of the flipping frame are the same as in the original translating frame. This shows again that it is not only the end positions that count.

**Movie 19.** 3-D rotation.

**Table 19.** Responses (out of 30).

		
Does the red flash appear to the right of the blue flash?	1	Yes.
	1	Yes, but only after a while
	3	Not so much.
	25	No.

#### When the probe is continuous

Here, the frame moves back and forth in its normal fashion (Movie 20), but when the probes are on continuously (rather than flashed), 93% of observers report little or no visible displacement of the continuous probes (Table 20). Seven percent report seeing some shift. The continuous probes alternate with the flashed probes to show the difference. The stationary probes were Duncker's (1929) original paradigm, although he used a single dot not two. With a continuous probe, the moving frame only produces a small, reversed motion in the probe (induced motion) when the frame's motion is so slow it is near the motion threshold (e.g. Nakayama & Tyler, 1978).

**Movie 20.** Continuous versus flashed probes.

**Table 20.** Responses (out of 30).

		
When the probes are steady, do they appear to move? Ignore the intervals where the probes flash.	1	Yes.
	1	Yes, but only after a while
	3	Not so much.
	25	No.

## Conclusions

Previously, the frame effect has been shown to be a remarkably strong illusion, separating aligned flashes by as much as the distance the frame travels (Özkan et al., 2021; Wong & Mack, 1981). Here, we explored a wide number of factors to evaluate their influence on the effect. The observations that we collected were only rudimentary subjective reports (e.g. yes, I see it) but they did help identify which stimuli gave strong effects and which gave weak, ambiguous, or no effects. In particular, we found that the frame effect was quite robust and held up for smoothly or abruptly displacing frames and for second-order as well as for first-order motion. It held up even when the frame changed shape or orientation between the end points of its travel. The frame's path could be nonlinear, even circular, although the more complex paths were less effective. Two examples using aperture effects (Movies 13 and 14) showed that the separation of the flashed tests was driven by the perceived motion, not the physical motion. Moreover, when there were competing, overlapping frames (Movie 12), the effect was determined by which frame was attended, although not all observers were able to switch attention between the frames.

These results suggest that the frame effect depends on initially tracking the moving frame. We assume that it is acquired and tracked by attention in the same fashion as a target in the multiple object tracking (MOT) task. The result with the overlapping frames supports the role of attention in this tracking – for many observers, the two overlapping frames did not cancel each other's effect; instead, the offset between the flashed tests depended on which frame was attended. The unattended frame then became ineffective. MOT and the frame effect also share an indifference to shape changes. Radical distortions in shape did not deter the frame effect in Movie 7 and shape changes of targets in MOT do not affecting performance, except when the shapes took on the properties of fluids (vanMarle & Scholl, 2003). Apparent motion shows a similar tolerance to shape changes (Kolers & Pomerantz, 1971; Kolers & von Grünau, 1976; Zhou, Zhou, Rao, Wang, Meng, Chen, Zhou, & Chen, 2003). In all these cases, it is a persisting “object” that is being tracked, one whose continuity depends primarily on spatiotemporal proximity rather than fixed shape. Kahneman, Treisman, and Gibbs (1992) addressed this same point with their concept of an “object file” as a transient representation of an entity that may change its properties over time but remains the same thing. In the case of the frame effect, this entity, the moving frame, is also serving as a reference for the location of events that take place in its neighborhood. Interestingly, this suggests that the frame effect could be a useful tool for evaluating the principle of continuity – offering an objective test based on the strength of the illusory shift of the flashed probes. The frame effect also holds promise for understanding mechanisms underlying visual stability where the displacement of the entire visual scene may act as a moving frame that stabilizes position as the eyes move.

We also found that there were a number of constraints that limited the effect. An isolated, static anchor near the flashes suppressed the effect but an extended static texture did not. When the frame's path kept one edge of the frame near the flash locations, the effect was reduced or eliminated. If the probes were continuous (as in Duncker, 1929) rather than flashed, the effect was abolished as well. Indeed, previous studies have shown that a moving frame influences a steady probe only when the frame's motion is very slow, much nearer motion threshold than the speeds used here (Nakayama & Tyler, 1978; Reinhardt-Rutland, 1988). The continuous probe may provide sufficient position information to overcome the influence of the frame, or perhaps the unmoving probe may group with the steady background beyond the moving frame, suppressing any effect of the frame.

**Figure 3.**
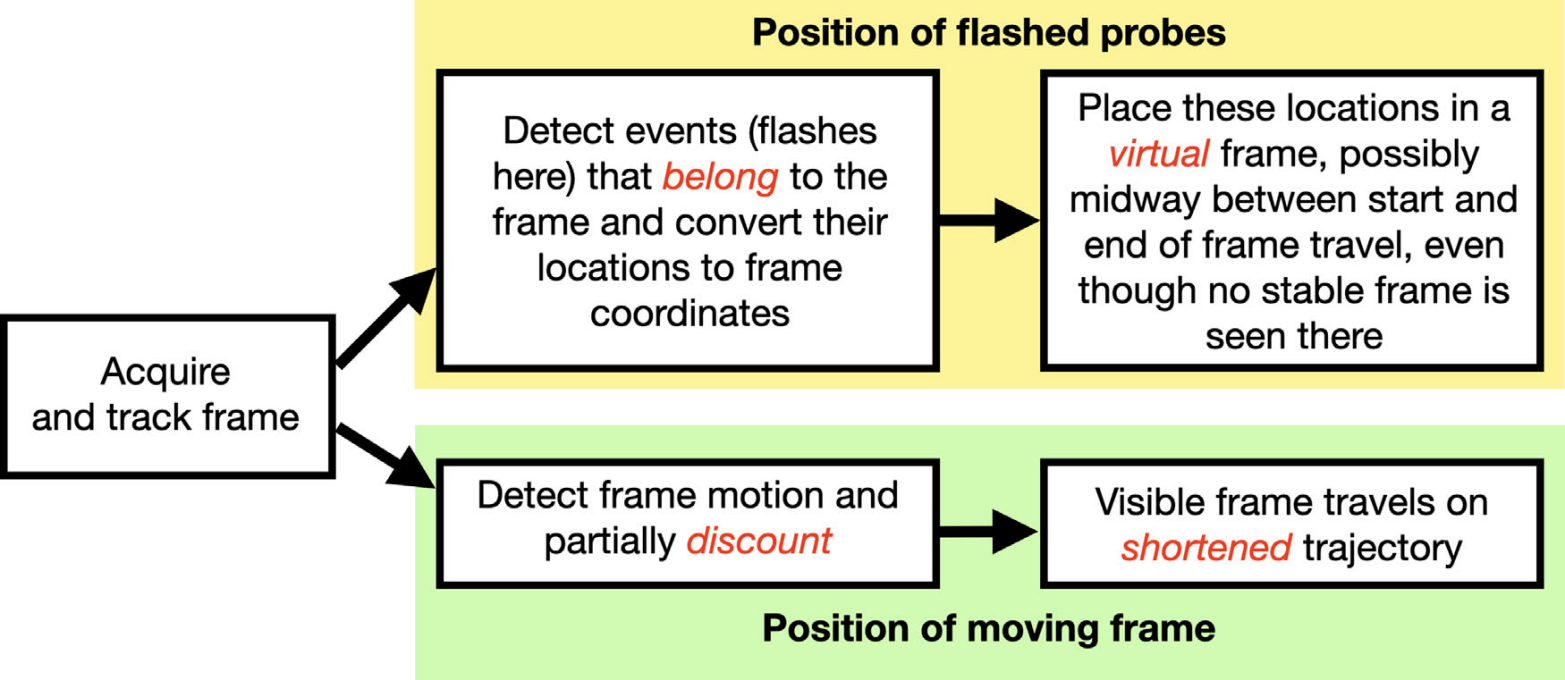
Proposed organization of the frame effect.

Our earlier article (Özkan et al., 2021) already established that the frame's motion can separate the perceived positions of the flashes by as much as the frame's travel—equivalent to being seen in frame coordinates (the locations in the frame where the probes were when they flashed) with the frame stationary. In this case, its motion would be completely discounted. But intriguingly, the frame is still seen to move quite well, albeit over a shortened path. Combined with these earlier results, our new observations lead us to propose a rough organization for the frame effect, laid out in Figure 3, where the frame's motion acts along two different pathways once the frame has been acquired and its motion tracked. The proposal of two separate pathways is not theoretically based, instead, it is required to deal with the paradoxical result of Özkan et al. (2021) that the frame's motion remains visible even though the flash displacements are consistent with a stationary frame.

In the first pathway, the upper path in Figure 3, the frame's motion acts on the positions of the flashes, contingent on the flashes belonging to or binding with the frame. For example, when attention switched from one frame to the other in Movie 12, the direction of the separation of the flashes switched as well, indicating that the flashes were seen in the context of, or belonging to, the attended frame. The test movies in this article did not explicitly manipulate belongingness so we cannot specify yet what elements are critical. However, the robust effect for the continuously rotating circle (Movie 11) does suggest that the synchrony of the flash presentation and the motion reversal is not required and also that some relative motion between the flash and the frame can be tolerated. In all the other examples, except the continuous probes (Movie 20), the frame's motion paused for the flash, but this is apparently not required for the grouping of the flashes and the frame as the illusory shifts were still seen in Movie 11 when the frame continued to move during the flash presentations.

Importantly, the large separations between the flashes measured by Özkan et al. (2021) matched their separations in frame coordinates—the locations the flashes had relative to the frame when they flashed. Moreover, this illusory separation was seen centered on the frame's path—as if the flashes were fixed in those locations relative to a static frame that was located in the middle of the path. However, this static frame was clearly not visible. We can think of this unseen frame as a virtual or a model frame, although we have no evidence yet of its existence.

The second pathway, the lower one in Figure 3, concerns the perception of the frame's motion. The frame does not come to a standstill as it should to be consistent with large flash separations, but its motion is nevertheless attenuated (Özkan et al., 2021). This “path shortening” has been reported for bounded motion trajectories before (Cavanagh & Anstis, 2013; Sinico, Parovel, Casco, & Anstis, 2009; Whitney, Murakami, & Cavanagh, 2000) and is apparent here again.

The observations here and the rough outline of how frames work in Figure 3 suggest several new directions for understanding the frame effect. These build on the nearly 100 years of research on the effect of frames on visual perception but bring new questions. How is the visual scene decomposed into hierarchical sets of dynamic frameworks? What are the limits to the changes of the frame that still maintain its continuity? What properties from each frame are inherited by the elements belonging to the frame? What determines when a test flash “belongs” to the frame? The remarkable strength of the frame effect on perceived location also suggests that it can serve as a “workhorse” tool, a visual equivalent to the Stroop task, that can be used to examine the details of how vision encodes the dynamic scenes unfolding in front of us.
